# Editorial: Chemical reactivity and solution structure

**DOI:** 10.3389/fchem.2023.1293697

**Published:** 2023-09-26

**Authors:** Leonid O. Kononov, Koichi Fukase, Nikolai F. Bunkin

**Affiliations:** ^1^ Laboratory of GlycoChemistry, N. D. Zelinsky Institute of Organic Chemistry, Moscow, Russia; ^2^ Department of Chemistry, Osaka University, Osaka, Japan; ^3^ Department of Fundamental Sciences, Bauman Moscow State Technical University, Moscow, Russia

**Keywords:** glycosylation, diluted aqueous solutions, THz spectroscopy, molecular dynamics (MD), antibodies, DFT, QTAIM, explicit solvation

Among factors determining the pathway and the outcome of a chemical reaction, only some depend on the structural and electronic features of the molecules of reactants, which are now considered to be well understood. This is not the case for the “environmental variables” (Chatterjee et al., 2018) many of which can be traced back to the structure of reaction solutions (Kononov, 2015). Importantly, many solutions of low molecular weight substances were found to be structured (inhomogeneous) at the nano and meso levels most likely due to the “solvophobicity driven mesoscale” structuring (Rak and Sedlák, 2023) induced by small quantities of “solvophobic admixtures” (Kononov, 2015; Rak and Sedlák, 2023) that are present in most “research-grade compounds of p.a. purity and even after special in-lab purification procedures” (Sedlák and Rak, 2014) since “no truly pure chemicals exist” (Rak and Sedlák, 2023).

For these reasons, understanding chemical reactivity in solutions requires much more than knowledge of molecular structures of reactants; moreover, we must go far beyond the present knowledge of specific solvent and solvation effects related to starting compounds, intermediates and transition states that emerge along the reaction pathway. The real reacting species in solutions seem to be not just individual molecules but more complex supramolecular entities that, generally, comprise both solute and solvent molecules. It appears that only detailed knowledge of the structure of reaction solution at the nano- and mesoscale level could reveal novel effective means for modulation of chemical reactions and processes. We believe that further progress in understanding chemical reactivity could be only achieved if we enlarge and deepen our knowledge of the structure of reaction solutions.

This Research Topic is a first attempt to gather a series of important works by the leading experts on the subject and to provide a balanced Research Topic of theoretical and experimental studies of structure of solutions and chemical reactivity.

The Research Topic includes one mini review article, and three original research articles, which come from Japan, Mexico, Germany, and Russia.

The mini review by Ishiwata et al. covers recent advances in the development of 1,2-*cis* selective glycosylations, including glycosyl donor modification, choice of reaction conditions, and activation methods for inter- and intra-molecular glycosylation. Of special importance for the current Research Topic is the use of inter-molecular interactions, such as hydrogen bonding, between the molecules of reactants to achieve the desired stereochemistry. A well-known but little understood influence of concentration of reactants on stereochemical outcome of glycosylation is also covered.

The importance of concentration for performing chemical reactions is already realized, and for this reason, it is not unsurprising that two articles are devoted to studies of the structure of solutions of various concentrations with special emphasis on ultra-dilute solutions.

In the article by Ryzhkina et al. self-organization in solutions of doxorubicin (Dox), a highly effective cytostatic antibiotic, and impact of Dox diluted aqueous solutions (calculated concentrations from 1·10^−20^ to 1·10^−4^ M) on hydrobionts are studied by a variety of physicochemical methods. It is established that aqueous solutions of Dox are dispersed systems which rearrange their dispersed phase measuring hundreds of nm in size (nanoassociates) upon dilution, followed by concerted changes in nanoassociates’ parameters (size and ζ-potential) and properties of systems, as well as their bioassay results. Electron microscopy results confirm and complement the light scattering data indicating the existence of nanoassociates in dilute Dox solutions.

Unusual properties of ultra-dilute protein (antibodies) solutions, which demonstrate an altered antigen-antibody specificity, are studied in the article by Woods using a combination of THz spectroscopy measurements with molecular dynamics simulations, which successfully reproduce the observed signatures from experimental measurements. The author has uncovered that the reorganization of the sample surface residue dynamics at the solvent-protein interface leads to both structural and kinetic heterogeneous dynamics that ultimately create interactions that enhance the binding probability of the antigen binding site. The results obtained indicate that the modified interfacial dynamics of antibodies to interferon gamma and to the interferon gamma receptor 1 are directly associated with alterations in the complementarity regions of the distinct antibodies that designate both antigen-antibody affinity and recognition.

Computer modeling of solvation is a widely used approach to explain and predict chemical reactivity in solutions. When the energies of solute-solvent and solvent-solvent interactions are close in magnitude, invoking explicit solvation model is required. However, it is not obvious where the explicit solvent molecules should be located. In their paper, Hernández-Lima et al. suggest their answer to this fundamental question in the implementation of explicit solvation. The authors performed NMR experiments which allowed them to determine the number of solvent molecules around the solute molecule which belong to the first coordination sphere. DFT and QTAIM calculations revealed interaction between the solute molecule and the solvent molecules from this solvent sphere. It was possible to determine how the first solvent sphere around a solute molecule modulates the solute conformation and selectivity of a chemical reaction involving the solute.

Summarizing, this Research Topic is a small but essential step towards a more comprehensive understanding structure of solutions and chemical reactivity. We hope that it will stimulate other researchers to get involved in this exciting area of scientific research.

We cannot not finish this short preface without acknowledging all the authors and reviewers who have contributed to this Research Topic. We also want to thank all the editorial assistants. There would not be such a nice Research Topic without your kind cooperation and great efforts.
